# Noninvasive SARS-CoV-2 detection using a low-cost electronic nose

**DOI:** 10.1016/j.bjid.2026.105805

**Published:** 2026-03-21

**Authors:** Gabriel Fialkovitz, Pedro Lobo Sousa, Amanda Miyuki Hidifira, Gustavo Henrique Pereira Boog, Bruno Montico Costa, Wellington Belarmino Gonçalves, Mariana Martins De Oliveira Netto, Alessandra Luna-Muschi, Pablo Munoz Torres, Lucas Augusto Moyses Franco, Ester Cerdeiro Sabino, Silvia Figueiredo Costa, Jonas Gruber, Anna Sara Levin

**Affiliations:** a Universidade de São Paulo, Faculdade de Medicina, Departamento de Infectologia, São Paulo, SP, Brazil; b Universidade de São Paulo, Faculdade de Medicina, Hospital das Clínicas, Unidade de Controle de Infecção Hospitalar, Instituto do Coração (InCor), São Paulo, SP, Brazil; c Universidade de São Paulo, Instituto de Química, Departamento de Química Fundamental, São Paulo, SP, Brazil

**Keywords:** SARS-CoV-2, COVID-19, Electronic nose, Noninvasive detection

## Abstract

The COVID-19 pandemic highlighted the urgent need for rapid and accurate SARS-CoV-2 detection. Current diagnostic methods often suffer from discomfort, slow results, and limited accuracy in early infection stages. This study proposes a solution: a noninvasive, rapid, and accurate detection approach for point-of-care settings using a metal-oxide-sensor-based electronic nose. This innovative electronic nose uses an array of off-the-shelf gas sensors. These sensors detect and analyze the volatile organic compounds present in saliva and exhaled breath, which change based on the presence of the SARS-CoV-2 virus. We evaluated the discriminatory power of the electronic nose using a suite of machine learning algorithms, specifically K-Nearest Neighbors (KNN), Support Vector Machine (SVM), Neural Networks (NN), and Random Forest, to differentiate between SARS-CoV-2 infected and non-infected samples. Accuracy metrics ranged from 76% to 89% for exhaled breath samples and from 75% to 86% for saliva samples. Optimal accuracy was achieved with the KNN algorithm, yielding an Area Under the Curve (AUC) of 0.861 (95% CI 0.825‒0.897) for saliva and 0.895 (95% CI 0.850‒0.940) for exhaled breath. These results support the feasibility and proof-of-concept performance of a low-cost electronic nose for SARS-CoV-2 detection in a real-world hospital cohort.

## Introduction

The global COVID-19 pandemic, caused by SARS-CoV-2, has had far-reaching consequences for both human health and the global economy. Accurately detecting the viral pathogen in asymptomatic carriers and symptomatic patients, along with developing effective therapeutic treatments, have presented significant challenges for healthcare providers.^1^

SARS-CoV-2 detection primarily relies on three methods: Nucleic Acid (NA)-based testing, antibody-based testing, and antigen-based testing. Quantitative real-time Reverse Transcription Polymerase Chain Reaction (rRT-qPCR) is considered the gold standard for COVID-19 testing due to its reliability, sensitivity, and specificity.^2^ However, PCR-based technologies are constrained by delivery time, typically taking 1 to 2 days after sampling. Furthermore, the high cost of equipment and personnel for implementing molecular diagnostic techniques, coupled with the ongoing need for reagents and materials during sample processing, hinders accessibility in low-resource settings.^3^

Mass collection of samples for large-scale testing also encounters challenges, as it relies on semi-invasive methods such as nasal and oropharyngeal swabs. These procedures can cause discomfort, lead to low adherence in children, and introduce variability in technique. This has created an urgent need for new noninvasive detection approaches capable of rapidly delivering accurate results, particularly in Point-of-Care Testing (POCT) scenarios.^4^

Molecular and antigen-based diagnostic methods, while highly accurate, are often limited in large-scale screening due to their high cost, infrastructure requirements, and the need for trained personnel. Because of these challenges, alternative sample types such as saliva have gained attention as reliable, non-invasive diagnostic specimens for SARS-CoV-2 detection. Several studies support this approach. For example, a Brazilian study demonstrated high agreement between saliva-based rRT-qPCR and conventional nasopharyngeal/oropharyngeal swab testing, while the GeneXpert Xpress SARS-CoV-2 assay showed comparable performance when applied to saliva samples, even at lower cycle threshold values.^5^^,^^6^ These findings highlight the potential of saliva to simplify sample collection, reduce discomfort, and mitigate occupational exposure risks for healthcare workers. Nevertheless, despite these advantages, challenges related to cost, reagent availability, turnaround time, and logistical constraints persist, particularly in resource-limited settings, underscoring the need for alternative diagnostic strategies that are rapid, scalable, and noninvasive.

In this context, diagnostic approaches based on the detection of Volatile Organic Compounds (VOCs) have emerged as a promising complementary strategy. Electronic nose (e-nose) instruments are capable of analyzing complex mixtures of VOCs derived from biological samples such as exhaled breath or saliva, generating characteristic response patterns that reflect underlying physiological and pathological states. E-nose technologies have been successfully applied across a wide range of medical conditions, including infectious diseases, genetic disorders, and noninfectious diseases such as asthma, cancer, cardiovascular disease, and diabetes.^7^, ^8^, ^9^, ^10^, ^11^ By leveraging multisensor arrays and pattern recognition algorithms, these systems can discriminate disease-associated VOC signatures without the need for specific molecular targets.^12^

Despite their diagnostic potential, most commercially available electronic nose platforms remain costly and require specialized infrastructure, limiting their adoption in low-resource environments. Furthermore, when applied to SARS-CoV-2 infection, the interpretation of VOC-based diagnostic signals must be situated within the broader clinical and virological context of the disease.

SARS-CoV-2 infection encompasses a heterogeneous spectrum of disease phenotypes, ranging from asymptomatic and mildly symptomatic presentations to severe viral pneumonia and systemic inflammatory disease. Across this spectrum, viral kinetics exhibit marked interindividual and temporal variability, with differences in peak viral load, timing of viral replication, symptom onset, and duration of viral shedding in both respiratory secretions and saliva. Seminal clinical virology studies have demonstrated that these kinetic patterns are dynamic and stage-dependent, directly influencing diagnostic sensitivity across biological matrices and over the course of infection.^13^, ^14^, ^15^

These aspects are particularly relevant for breath- and saliva-based diagnostic approaches, as Volatile Organic Compound (VOC) profiles may reflect not only viral presence but also host metabolic, inflammatory, and immune responses that differ across disease phenotypes and evolve in parallel with viral kinetics. Consequently, VOC-based signals are likely to vary according to disease stage, symptom severity, and host response rather than representing static markers of infection.

Within this complex clinical and virological landscape, the feasibility of low-cost electronic nose-based diagnostic strategies using noninvasive matrices warrants systematic evaluation, particularly in heterogeneous, real-world clinical settings.

This study therefore aims to evaluate the feasibility of employing a low-cost electronic nose for the noninvasive detection of SARS-CoV-2 infection using saliva and exhaled oral breath samples.

## Materials and methods

### Study design

This proof-of-concept feasibility study was conducted at the Hospital das Clínicas of the University of São Paulo Medical School (HC-FMUSP) between May and October 2021. The study included patients admitted for any reason to the emergency, inpatient, and intensive care units, all of whom had RT-PCR tests routinely collected upon admission. Additionally, healthcare professionals who underwent RT-PCR testing due to respiratory symptoms also participated. All participants provided informed consent before their involvement. The data collected was assessed in an aggregated manner to ensure the confidentiality of personal and clinical information. The study received approval from the Hospital das Clinicas’ Committee for Ethics in Research (CAAE: 43,880,521.1.0000.0068).

### Study population

Participants were divided into two groups based on their RT-PCR results: 1) SARS-CoV-2 positive and 2) SARS-CoV-2 negative. To ensure robust statistical analysis, we aimed for 90% statistical power at a 0.05 significance level. Based on a hypothesized area under the receiver operating characteristic curve (AUC) of 0.80 for the experimental group and 0.50 for the null hypothesis,^16^ sample size calculations indicated a minimum of 19 subjects per group. We used chi-squared tests and Analysis of Variance (ANOVA) to explore potential differences in baseline characteristics between the groups.

### Setup for sample collection

Patients admitted to the hospital's inpatient units were screened for study enrollment, with an active search for new patients conducted irrespective of their respiratory symptoms. Furthermore, healthcare professionals exhibiting respiratory symptoms and undergoing treatment at a specialized service for hospital staff were also assessed for inclusion. All patients who provided informed consent and were able to furnish saliva and exhaled breath samples were enrolled in the study.

To ensure safety during saliva and breath collection, researchers used comprehensive personal protective equipment, including coveralls, masks, gloves, and goggles. Besides breath collection, participants completed a brief questionnaire detailing symptom onset and type. Recorded data also included demographic factors like age, sex, smoking behavior, and the presence of comorbidities. All data was anonymized and tabulated for later analysis.

All study participants underwent SARS-CoV-2 RT-PCR testing, either as part of their routine hospitalization protocol or for clinical status evaluation within their hospital units. The Molecular Biology Laboratory of HC-FMUSP performed the RT-PCR analysis on nasopharyngeal and oropharyngeal swab samples.^17^ The Artus® Prep&Amp UM (QIAGEN, Hilden, Germany) was used for the RT-PCR test.

Saliva samples were collected using a drooling method as described in reference.^18^ Participants allowed unstimulated saliva to accumulate in their mouths for one minute before draining it into an inert 50 mL polypropylene Falcon tube, aiming for a minimum volume of 5 mL. For expired air collection, an inert polypropylene straw was used. Participants exhaled for 5‒10 seconds, repeating the process six times to create bubbles in a 0.9% saline solution, also contained in a Falcon tube. This technique captured volatile organic compounds that condensed in the liquid. After collection, all tubes were sealed for transport and remained sealed until electronic nose analysis. Samples were sent to an external laboratory and analyzed within 24 hours of collection, stored at 4‒8°C.

Before sample collection, patients rinsed their mouth with water for cleaning. However, we couldn’t ensure that they hadn’t consumed food or beverages prior to collecting saliva or expired air samples.

### Sensing apparatus

The homemade e-nose prototype incorporates an array of ten commercially available Metal-Oxide-Semiconductor (MOS) gas sensors: MQ2–MQ9, MQ135 (Winsen Electronics, China) and TGS2620 (Figaro, USA). These sensors were chosen due to their low cost and widespread availability, enabling the construction of an affordable electronic nose.^12^

The use of a broader sensor array was intended as an exploratory approach to evaluate sensor responsiveness and discriminatory potential in this proof-of-concept study, rather than to imply optimized chemical specificity.

The sensors are interfaced with an Arduino Mega open-source platform. This platform’s role is to digitize the sensors’ analog outputs, generating a table of reading times and electrical response values for subsequent graphical and mathematical analysis. Additionally, it controls the air pumps of the pneumatic system, which are described below.

**Fig. 1:**
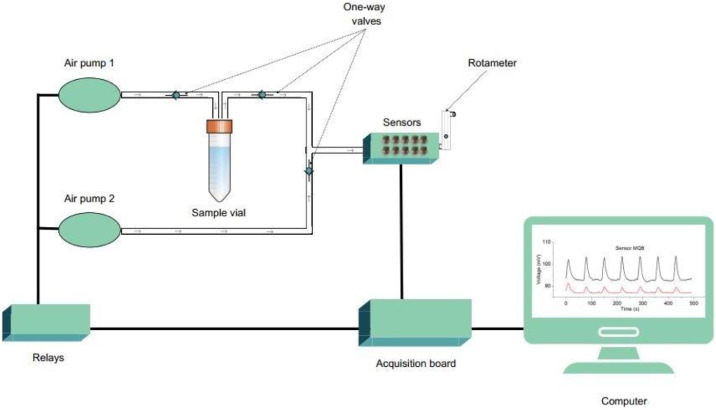
Schematic diagram of the electronic nose’s pneumatic flow system utilized for the diagnosis of SARS-CoV-2 infection.

Fig. 1 illustrates the e-nose’s pneumatic system. An exposure pump introduces the sample headspace into the sensors’ chamber. After the exposure period, this pump turns off and a recovery pump activates. The recovery pump delivers fresh air, free of volatile compounds, to the sensors’ chamber. This process cleans the sensors and prepares them for the next exposure cycle. A rotameter measures the system’s airflow. Retention valves (one-way valves) prevent the sample volatiles from reaching the recovery pump and stop cleaning air from entering the sample compartment. To assess sensor stability over time, ethanol-saturated air at 25°C was used.

The system connects to computers via USB, enabling users to customize parameters such as exposure and recovery times, number of cycles, and sampling rate. This enhances its adaptability and utility. The total cost of the system was approximately US$ 240.

During the measurements, a 5 mL conical tube of either saliva or saline solution was placed inside the e-nose’s sample chamber. Samples were kept at room temperature, and the airflow rate was maintained at 1 L.min^-1^. No further sample treatment was necessary. Sensor responses were collected over seven cycles, each consisting of a 10-second exposure and a 60-second recovery period. The exposure time was chosen to prevent sensor saturation, while the recovery time was set to allow the sensors to return to their original baseline readings with less than a 2% deviation, preventing prior measurements from affecting subsequent ones. The entire analysis took approximately 8.2 min.

### Data processing

Origin software (Origin Lab, Northampton, MA, USA) was employed for all statistical analysis and Principal Component Analysis (PCA) calculations. Classifier evaluation was conducted using Weka software developed by the University of Waikato in New Zealand. The data processing for this study involved three main steps: feature extraction, feature selection, and pattern recognition.

### Feature extraction

Normalization of raw data was achieved through the application of a fractional difference model (Eq. 1), which incorporates the Relative Response (RR). Here, R_max_ denotes the peak system response to the VOC pattern of the sample, and R_0_ refers to the initial baseline recording.

(1) $$RR=\frac{Rmax-R0}{R0}\;$$

### Pattern analysis

To capture the maximum variability within the data, PCA was used. PCA is a widely adopted multivariate analysis method that transforms data using orthogonal vectors based on variance. It is commonly applied for both dimension reduction and discrimination analysis.^19^

Alongside the graphic analysis, various machine learning classifiers were applied to classify the samples. Support Vector Machine (SVM) is a statistical machine learning algorithm effective for both regression and classification, especially with non-linear data. It works by constructing a hyperplane to optimally separate different classes. To overcome limitations with linear separation, the polynomial kernel is often used as an SVM classifier function for non-linear classification tasks. The Pearson VII function, inspired by the original, can also serve as a universal kernel replacement for widely used kernel functions.^20^ The Artificial Neural Network (ANN) is a prevalent artificial intelligence technique and an extensively employed model known as the Multilayer Perceptron. The classification process involved the utilization of a network of 20 hidden layers and 500 epochs.^21^ The K-Nearest Neighbors (KNN) algorithm is a popular non-parametric method; it assigns a data point to a class or predicts a value based on the majority vote or averaging of its K nearest neighbors in the feature space.^22^ Finally, Random Forest combines multiple decision trees by creating an ensemble of trees and considering different subsets of features, reducing overfitting, and increasing diversity.^23^

### Cross-validation

To optimize feature selection and pattern recognition, and to assess the performance of the proposed model, ten-fold cross-validation was used. This involved dividing the original dataset into ten subsets. Each subset served as a test set once, with the remaining subsets used for training. This process was repeated ten times, and the overall performance was calculated by summing the results from each fold.^24^^,^^25^ This approach ensures a robust model evaluation by reducing the potential bias that could result from a single train-test split.

### Evaluation of classification performance

To visually evaluate the e-nose’s ability to distinguish between the odor fingerprints of SARS-CoV-2 patients and control subjects, the PCA results were presented in a two-dimensional score plot. Additionally, the norm of the PC scores was calculated for each subject, and a one-way analysis of variance (ANOVA) was performed to compare the two groups.

To assess the model's diagnostic accuracy, three key metrics were evaluated: the area under the Receiver Operating Characteristic (ROC) Curve (AUC), sensitivity, and specificity. The AUC serves as an indicator of the model's overall performance. Sensitivity quantifies the proportion of actual positive cases that are correctly identified as such, while specificity measures the proportion of actual negative cases that a diagnostic test correctly identifies as negative. Collectively, these metrics provide insights into the model's capacity to accurately identify both positive and negative instances.

## Results

In total, 69 saliva samples were collected, 40 from SARS-CoV-2 subjects (35 of which were symptomatic) and 29 samples from the negative control group. Additionally, 46 exhaled oral breath samples were collected, comprising 28 from the SARS-CoV-2 positive group and 18 from the control group. Patient’s characteristics are presented in Table 1.

**Table 1:** Baseline characteristics of the 69-patient study cohort.

Characteristics	SARS-CoV-2 positive (40)	SARS-CoV-2 negative (29)	p-value
Age [years-mean (SD)]	57 (±16)	45 (±17)	**0.004**
Female [n (%)]	17 (44%)	23 (76%)	**0.007**
Smoking status [n (%)]	11 (28%)	4 (13%)	0.195
Hypertension [n (%)]	21 (54%)	10 (33%)	0.171
Diabetes [n (%)]	16 (41%)	5 (17%)	0.051
Obesity [n (%)]	9 (23%)	4 (13%)	0.396
Hypothyroidism [n (%)]	2 (5%)	0 (0%)	0.229
Neoplasm [n (%)]	4 (10%)	4 (13%)	0.589
Kidney disease [n (%)]	11 (28%)	4 (13%)	0.195
Cardiovascular disease [n (%)]	7 (18%)	4 (13%)	0.723
Lung disease [n (%)]	6 (15%)	3 (10%)	0.607
Liver disease [n (%)]	1 (3%)	1 (3%)	0.796
Immunodeficiency [n (%)]	15 (39%)	5 (17%)	0.080
Number of comorbidities [median (IQR)]	3 (1‒5)	2 (0‒4)	0.050

SD, Standard Deviation; IQR, Interquartile Range.

The control group consisted of 29 patients (6 males, 23 females), with an average age of 44±17 years. In contrast, the SARS-CoV-2 positive group had 40 patients (23 males, 17 females) and an average age of 56±16 years. Notably, fever, hypoxemia, dyspnea, and cough were the most frequently observed symptoms in the SARS-CoV-2 positive group at the time of sample collection (p < 0.005), as detailed in Table 2.

**Table 2:** Clinical profile of the 69-patient study cohort.

Symptoms	SARS-CoV2 positive (40)	SARS-CoV2 negative (29)	p-value
Fever [n]	23 (57.5%)	6 (20%)	0.003
Coryza [n]	14 (35%)	8 (27%)	0.577
Myalgia [n]	12 (30%)	8 (27%)	0.898
Dysgeusia [n]	4 (10%)	2 (7%)	0.682
Hypoxemia [n]	25 (62%)	6 (20%)	<0.001
Cough [n]	29 (65%)	12 (40%)	0.013
Odynophagia [n]	3 (8%)	6 (20%)	0.095
Anosmia [n]	3 (7%)	1 (3%)	0.498
Dyspnea [n]	27 (67%)	8 (27%)	<0.001

Initial inspection of the raw sensor data revealed that only the MQ5 and MQ6 sensors showed a response significantly above noise level. Both sensors are designed to detect Liquefied Petroleum Gas (LPG), with the MQ5 specifically sensitive to methane and the MQ6 to propane. Their datasheets (Winsen Electronics Technology Co., Ltd., 2018; Winsen Electronics Technology Co., Ltd., 2015), indicate a detection range of 300 to 10,000 ppm (0.03% to 1%) for both.

Analysis of the saliva samples *(*Supplementary Fig. S1) and exhaled oral breath samples (Supplementary Fig. S2) revealed noticeable differences in sensor responses between the SARS-CoV-2 and control groups.

Principal Component Analysis (PCA) for saliva and exhaled oral breath (Supplementary Figs. S3 and S4*)* demonstrated less effective group separation for exhaled oral breath when compared to saliva.

**Fig. 2:**
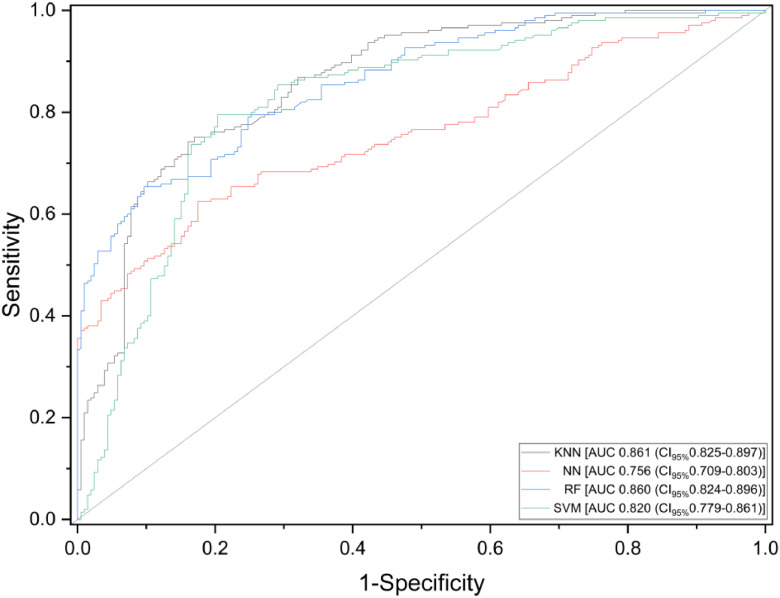
Receiver Operating Characteristic (ROC) curves of various machine learning algorithms ‒ K-Nearest Neighbors (KNN), Support Vector Machines (SVM), Artificial Neural Networks (ANN), Random Forest (RF) ‒ in detecting SARS-CoV-2 infection using saliva samples. Sensitivity is defined as the proportion of actual positive cases correctly identified, while specificity represents the proportion of actual negative cases correctly identified.

**Fig. 3:**
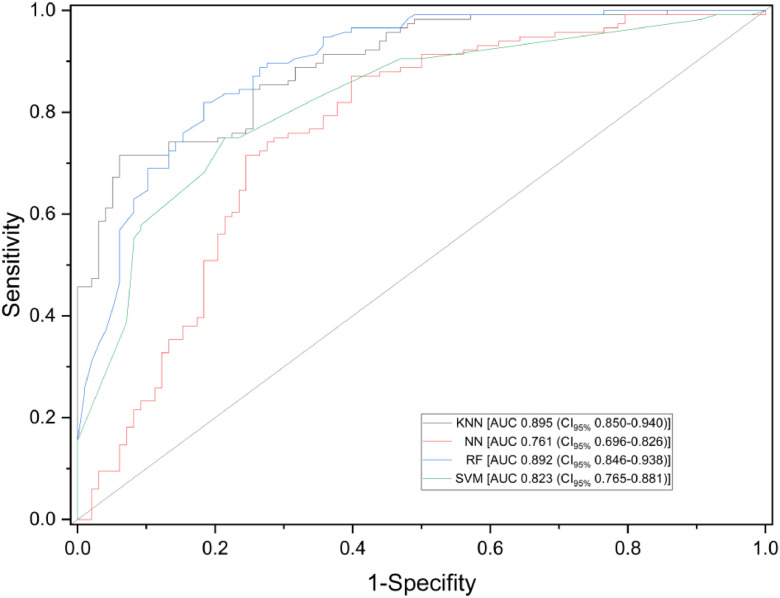
Receiver Operating Characteristic (ROC) curves of various machine learning algorithms ‒ K-Nearest Neighbors (KNN), Support Vector Machines (SVM), Artificial Neural Networks (ANN), Random Forest (RF) ‒ in detecting SARS-CoV-2 infection using exhaled breath samples. Sensitivity is defined as the proportion of actual positive cases correctly identified, while specificity represents the proportion of actual negative cases correctly identified.

Diverse outcomes were obtained when applying the algorithms KNN, SVM, ANN and RF to both saliva and exhaled breath samples (Fig. 2, Fig. 3). For saliva sample analysis, KNN and RF achieved 86% accuracy. SVM performed slightly less accurately at 82%, while ANN showed the lowest performance at 75%. Conversely, in the exhaled breath sample analysis, KNN and RF again led with 89% accuracy, followed by SVM at 82%, and ANN with 76%.

## Discussion

This study demonstrated the feasibility of using a low-cost electronic nose to differentiate SARS-CoV-2 infected patients from negative controls by analyzing saliva and exhaled breath samples. Different algorithms were tested, all yielding accuracy rates above 75%. Specifically, K-Nearest Neighbors (KNN) and Random Forest (RF) algorithms demonstrated comparable performances, achieving 86% accuracy for saliva samples and 89% for exhaled breath samples. However, these findings should be interpreted as preliminary, given the exploratory nature of the study and the characteristics of the enrolled population.

Receiver Operating Characteristic (ROC) curves were used to summarize overall discriminatory performance across the evaluated algorithms. No clinically defined decision thresholds were derived, and no direct comparisons were performed with existing rapid diagnostic tests. Consequently, the presented performance metrics should be interpreted as descriptive of discrimination rather than as indicators of clinical decision-making.

Previous studies investigating electronic nose technologies for SARS-CoV-2 detection have primarily focused on exhaled breath analysis, reporting overall accuracies typically ranging from approximately 80% to 95%, often using proprietary or custom-built sensor arrays under controlled or semi-controlled experimental conditions.^26^, ^27^, ^28^ In several of these investigations, higher performance metrics were achieved in relatively homogeneous study populations, frequently with standardized breath collection protocols and limited representation of patients with concurrent respiratory conditions.

By contrast, the present study evaluated both exhaled breath and saliva samples using a low-cost metal oxide semiconductor sensor array and open-source analytical tools in a real-world clinical environment. While the discriminatory performance observed in this study falls within the lower-to-middle range of previously reported accuracies, important methodological differences likely contribute to these variations. These include the use of broadly sensitive, non-specific MQ-series sensors rather than proprietary arrays, bedside sampling without environmental controls, smaller and imbalanced group sizes, and a study population enriched with hospitalized patients and symptomatic healthcare workers, many of whom presented with other respiratory illnesses.

In addition, differences in analytical pipelines ‒ such as feature extraction strategies, classifier selection, and validation approaches ‒ as well as heterogeneity in disease severity and symptom duration across studies further limit direct quantitative comparison of performance metrics.^4^^,^^29^ Within this heterogeneous landscape, the present findings demonstrate that performance levels comparable to those reported in breath-based electronic nose studies can be achieved using low-cost hardware and accessible platforms, albeit within an exploratory framework that warrants cautious interpretation and further validation.

Current COVID-19 diagnostic techniques are often limited by high costs, reliance on specialized personnel (especially for molecular biology methods), and the use of expensive single-use consumables, such as those required for antigen detection. In contrast, analyzing VOC using electronic noses offers a promising and practical alternative, particularly for point-of-care applications. However, the widespread adoption of this technology has been hindered by the prohibitive cost of commercially available electronic nose devices, which limits their accessibility in resource-constrained environments. Nevertheless, the development of electronic noses employing low-cost sensors and readily available open-source electronic prototyping platforms like Arduino can overcome these barriers, significantly promoting the broader adoption and accessibility of this innovative diagnostic technology. Our study showed that only two out of ten sensors yielded satisfactory responses, suggesting that further investigation into alternative sensors will likely produce even more promising results.

It is important to note that the study was conducted in a real-world setting, where patients in the SARS-CoV-2 negative group frequently presented with other respiratory illnesses. Despite these co-occurring conditions, differentiation between the control group and the COVID-19 group was observed. Some overlap between groups, particularly in exhaled breath samples, was evident, as illustrated in the PCA analyses (Supplementary Fig. S3 and S4). However, the study population consisted primarily of hospitalized patients and symptomatic healthcare workers, which may have overrepresented individuals with moderate-to-severe disease and limited the inclusion of asymptomatic or mildly symptomatic community cases. As a result, the diagnostic performance observed may not be directly generalizable to population-based screening scenarios.

As the study was conducted during the height of the COVID-19 pandemic, a comprehensive comparison between the SARS-CoV-2 groups and other respiratory viruses was not feasible. Future applications of this technology should explore its efficacy in identifying the etiology of other viral respiratory diseases to fully assess its diagnostic accuracy.

A potential limitation of the study was that sample collection occurred at the bedside across various hospital settings, which may have introduced interference from background environmental volatile components. Although differentiation between groups was observed under these real-world conditions, the study did not include environmental controls or quantitative assessments of ambient VOCs, and therefore the potential impact of environmental interference on sensor specificity and measurement reproducibility cannot be fully excluded. Within this context, the observed differentiation should be interpreted as feasible under bedside conditions rather than as evidence of environmental robustness. In addition, although the study was powered to evaluate AUC discrimination, the relatively small sample size, with a minimum of approximately 19 subjects per group and a total of 69 samples, limits the generalizability of the findings, the precision of performance estimates, and the ability to adjust for potential confounding variables. Therefore, these results should be interpreted as exploratory, and larger multicenter studies will be necessary to confirm the diagnostic performance observed.

Although demographic characteristics, comorbidities, smoking status, and clinical features were collected, the sample size did not allow for robust multivariable or stratified performance analyses, and the potential influence of these factors on diagnostic performance could not be formally assessed. Additionally, the study did not evaluate whether diagnostic performance varied according to symptom duration, disease severity, or RT-PCR cycle threshold, which may be relevant for assessing early diagnostic utility but could not be reliably explored within the scope and sample size of this exploratory study.

Regarding hardware limitations, while sensor stability was preliminarily assessed using ethanol-saturated air under controlled conditions, long-term sensor drift, inter-sensor variability, and cross-reactivity with other respiratory pathogens were not systematically evaluated in this study.

The observation that MQ5 and MQ6 sensors contributed most strongly to discrimination should be interpreted as an empirical finding rather than as evidence of specific chemical selectivity. Volatile Organic Compound (VOC) signatures have been widely reported as downstream products of host metabolic activity, inflammatory responses, oxidative stress, and interactions with microbial communities in the respiratory tract.^30^^,^^31^ These sensors are broadly sensitive to hydrocarbons and LPG-related compounds and likely capture composite VOC patterns rather than individual analytes. In the context of SARS-CoV-2 infection, alterations in breath and saliva VOC profiles may be linked to host metabolic dysregulation, inflammatory responses, oxidative stress, and downstream effects on lipid peroxidation and cellular respiration.^31^^,^^32^ These processes may result in increased production or altered proportions of hydrocarbons, aldehydes, ketones, and related volatile metabolites that have been associated with COVID-19 and other respiratory conditions.^29^ In this context, the predominance of MQ5 and MQ6 may reflect their ability to capture such composite changes in VOC mixtures. Nevertheless, despite observable separation between groups, overlap remained ‒ particularly in exhaled breath samples ‒ suggesting imperfect discrimination and a potential risk of misclassification in real-world applications. Furthermore, although an array of ten sensors was employed, the fact that only two sensors demonstrated meaningful responses may indicate redundancy within the current sensor configuration and limited chemical specificity. Importantly, the present study was not designed to identify individual VOCs or directly interrogate biochemical pathways, and any mechanistic interpretation remains speculative, requiring targeted analytical approaches such as gas chromatography-mass spectrometry for further elucidation of specific biomarkers.

From a translational perspective, the present findings should be interpreted within the scope of a proof-of-concept study. While the results demonstrate feasibility under research conditions, broader validation across multiple centers and epidemiological contexts will be required to assess generalizability, reproducibility, and operational performance. These considerations are essential for future studies aiming to bridge the gap between exploratory research and potential clinical application.

## Conclusion

Previous studies have already demonstrated the potential use of electronic nose technology for COVID-19 diagnosis.^26^, ^27^, ^28^ However, these studies relied on expensive, commercially available products. Our work suggests that high diagnostic accuracy in detecting SARS-CoV-2 infected samples may be achievable using simple, user-friendly, and cost-effective equipment; however, these findings are preliminary and require confirmation in broader populations, including asymptomatic or mildly symptomatic community cases. Since completing this study, we have developed even lower-cost equipment based on these principles, which is currently undergoing evaluation and algorithm development. Taken together, these results support the feasibility of this approach as a potential alternative for point-of-care diagnosis of infections, particularly in low-resource settings.

## Declaration of the use of generative artificial intelligence

In this scientific work, generative artificial intelligence (AI) has not been used.

## Data availability statement

The raw data supporting the conclusions of this article will be made available on request by e-mail to the corresponding authors.

## Fundings

This work was supported by the 10.13039/501100003593Conselho Nacional de Desenvolvimento Científico e Tecnológico (CNPq) through grants 140100/2020–2 (P.S.L), 310048/2022 (J.G.) and 165186/2015–1 (W.B.G.).

## Conflicts of interest

The authors declare no conflicts of interest.

## Appendix Supplementary materials

Image, application 1
