# Supplementary material for: Noninvasive SARS-CoV-2 detection using a low-cost electronic nose
Source: Braz J Infect Dis. 2026 Mar 21;30(2):105805. doi: 10.1016/j.bjid.2026.105805 (PMC13069424; doi:10.1016/j.bjid.2026.105805)

**BJID-D-25-00298**

**Supplementary Material**

**Figure S1** Responses of MQ5 and MQ6 sensors to volatile components from saliva samples of SARS-CoV-2 positive individuals and negative controls.


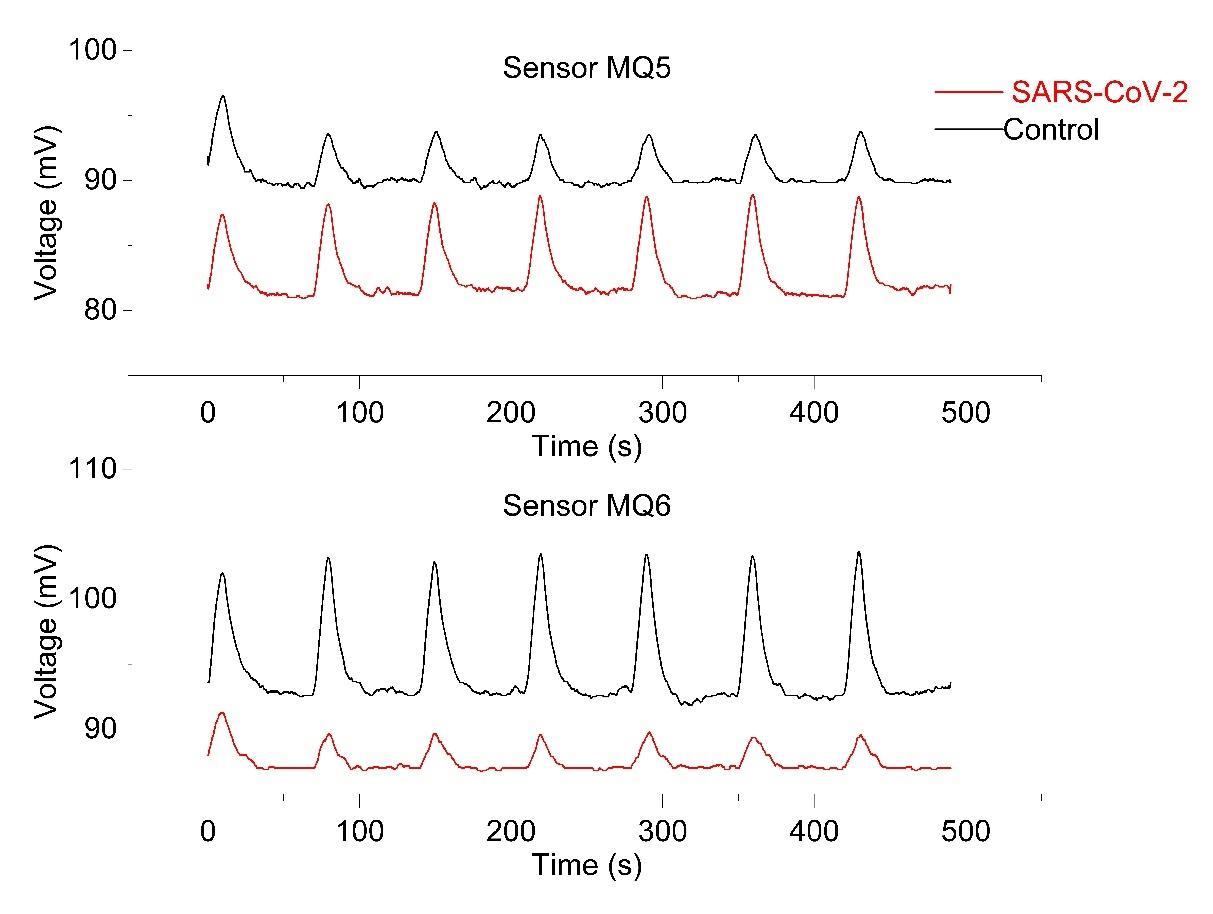


**Figure S2** Responses of MQ5 and MQ6 sensors to volatile components from exhaled breath samples of SARS-CoV-2 positive individuals and negative controls.


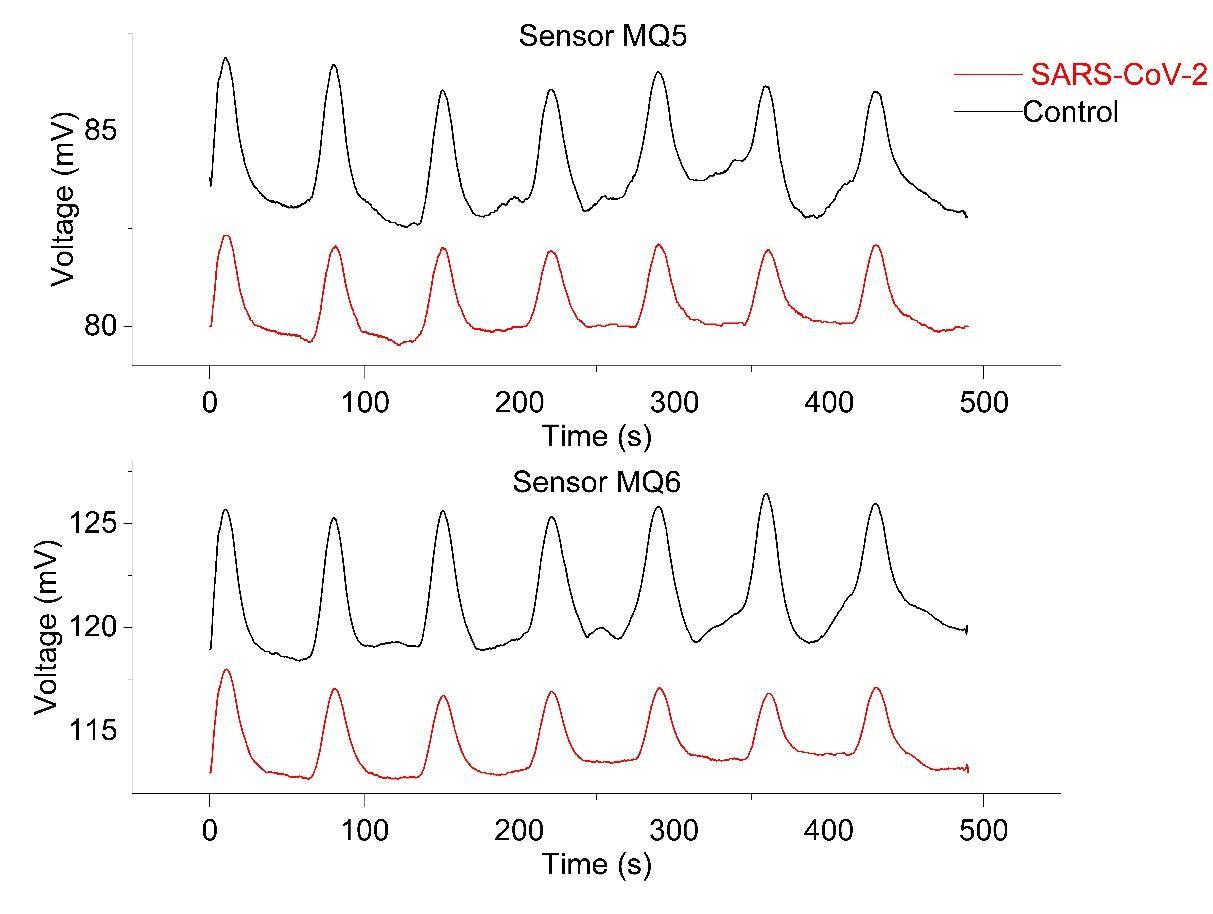


**Figure S3** Principal Component Analysis (PCA) score plot for the saliva samples for the detection of infection by SARS-CoV-2. The ellipse encompasses 95% of the data.


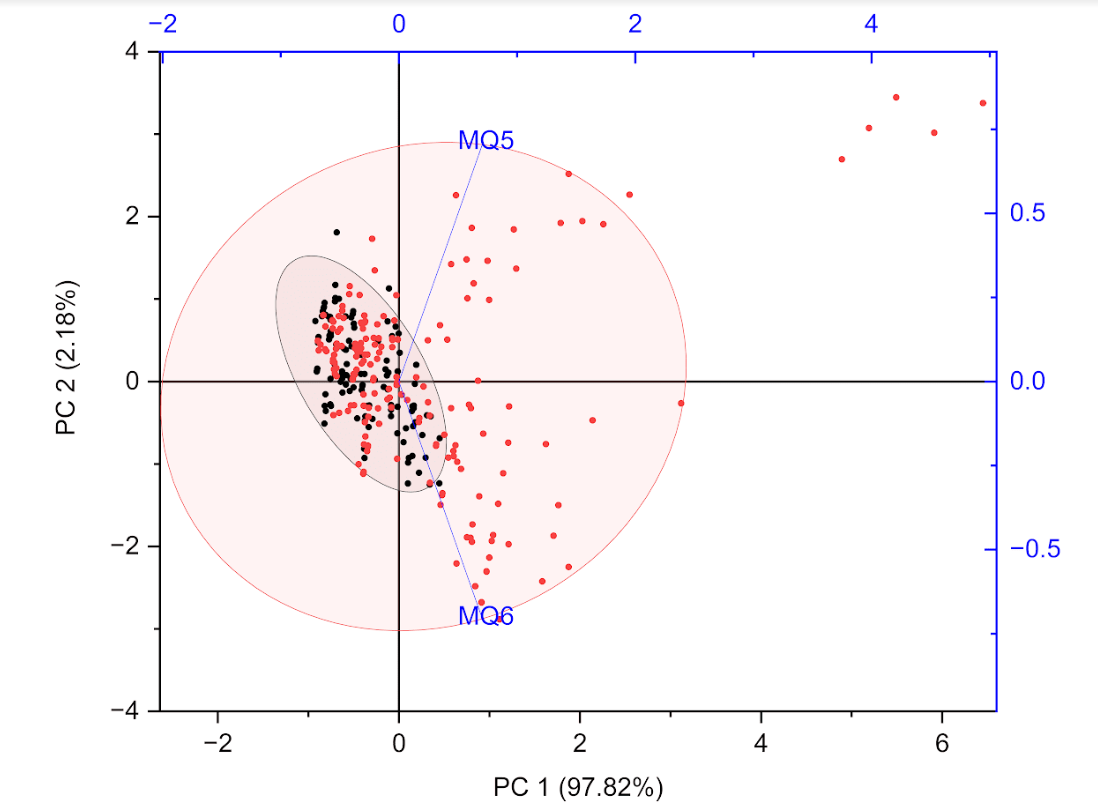


**Figure S4** Principal Component Analysis (PCA) score plot for the exhaled breath samples for the detection of infection by SARS-CoV-2.


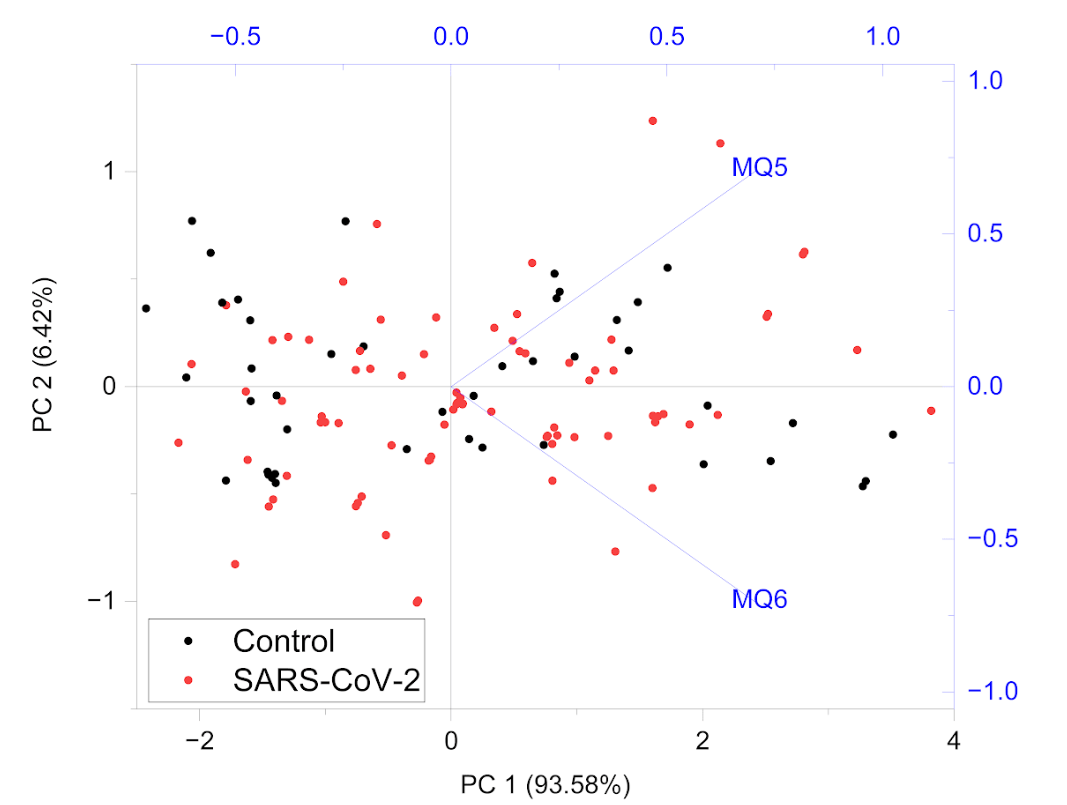

Supplement: Supplementary file 1 [file mmc1.docx]
